# Oil-soluble contrast medium bathing attenuated endometrial inflammation and improved endometrial receptivity in women with recurrent implantation failure: a descriptive study

**DOI:** 10.1186/s12905-024-03160-6

**Published:** 2024-06-05

**Authors:** Qiuyan Huang, LinIing Mo, Junli Wang, Aiping Qin

**Affiliations:** 1 Department of Obstetrics and Gynecology, The First Affiliated Hospital of Jinan University, Guangzhou, 510632 Guangdong China; 2 Key Laboratory of Metabolic Diseases of Baise, Affiliated Hospital of Youjiang Medical University for Nationalities, Baise, 533000 Guangxi China; 3 The First Affiliated Hospital of Guangxi Medical University, Nanning, 530022 Guangxi China; 4 Maternal and Child Health Hospital of the Guangxi Zhuang Autonomous Region, Nanning, 530028 Guangxi China; 5 Youjiang Medical University for Nationalities, Baise, 533000 Guangxi China

**Keywords:** Oil-soluble contrast, Failed embryo transfers, Frozen-thawed embryo transfer, Window of implantation

## Abstract

**Background:**

The oil-soluble contrast medium used in hysterosalpingography has been shown to have a fertility-enhancing effect, but the underlying mechanism is unclear, especially regarding the role of window of implantation (WOI). This study aimed to assess the endometrial immunological impact of the WOI before and after bathing with the oil-soluble contrast medium in women with recurrent implantation failure (RIF).

**Methods:**

This descriptive study involved two medical centers between December 18, 2019, and December 30, 2020. We included infertile women who underwent three or more transfer cycles, cumulative transplantation of at least four high-quality cleavage-stage embryos or three high-quality blastocysts without clinical pregnancy, and high-quality frozen embryos that were still available for implantation. Patients received 5 ml of ethiodized poppyseed oil bathing, endometrial biopsy around bathing, and frozen-thawed embryo transfer (FET) within four menstrual cycles after bathing. Patients were excluded if failure to complete anyone. Data on the baseline characteristics and clinical data of the FET cycles were collected, and endometrial biopsy specimens were collected in the luteal phase before and after bathing and subjected to immunohistochemistry. The number of CD56 and CD138 positive cells and H-score of expression of ανβ-3 and HOXA10 in endometrium were collected.

**Results:**

Thirty-four patients were initially enrolled in the study; ultimately, twelve patients with a median age of 32.5 years (range 27–40 years) completed the research. The median number of embryo transfer cycles was three (range 3–8). A total of 4 of 12 women (33.33%) were diagnosed with chronic endometritis before oil-soluble contrast bathing. After bathing, the median numbers of CD138-positive cells in endometrium decreased from 0.75 (range 0–13.5) to 0.65 (range 0–6), *P* = 0.035; additionally, the H-score of expression of ανβ-3 in endometrium increased from 148.50 ± 31.63 to 175.58 ± 31.83, *P* < 0.001. The thickness of the endometrium also significantly increased (8.90 ± 1.45 mm vs.10.11 ± 1.98 mm, *P* = 0.005). However, no consistent changes were found in the expression of CD56 and HOXA10 in the endometrium. Five patients experienced biochemical pregnancies (41.67%), four had clinical pregnancies (33.33%), and three achieved live births following oil-soluble contrast bathing (25%).

**Conclusions:**

These results suggest that oil-soluble contrast medium bathing decreased CD138-positive cells and upregulated expression of ανβ-3 during WOI in patients with RIF. This histological impact of endometrium may result in enhanced fertility during FET cycles. Investigating the ability of intrauterine bathing with lower-dosage oil-soluble contrast to improve pregnancy in the RIF population is warranted.

## Introduction

Clinical experience of more than five decades, including multiple randomized clinical trials (RCT), suggested that oil-based contrast medium has a more significant effect than water contrast in hysterosalpingography (HSG) [1–3]. The fertility-enhancing effect of oil-based contrast medium generally lasts six months [1, 2, 4] or even up to 5 years [5]. The subgroups of infertile women, including those with fertility for women with normal tubal patency and unexplained and endometriosis-related infertility [4, 6, 7], were the primary beneficiaries. Advanced age, ovulation disorders, or high risk for tubal pathology, who may benefit, have also attracted increased new attention [8]. However, the underlying mechanism of the fertility-enhancing effect induced by HSG with oil-based contrast medium remains unclear.

Oil-based contrast agents, unlike water-based ones, are mainly made of iodine-based fatty acids from poppy seed oil, and the oil is not miscible in water [9], resulting in high viscosity and longer-lasting effects in the pelvic and uterine cavity [10]. Some hypotheses indicate that the mechanism occurs in the fallopian tube [11], the peritoneum [12], or the endometrium [13]. It is thought to effectively clear debris from the fallopian tubes that might impede fertility, and the significant interaction between pain in oil-based contrast medium used in HSG and ongoing pregnancy suggests that debris or mucus plugs from the proximal part of otherwise regular fallopian tubes are flushed by increasing intrauterine pressure [11]. Izumi, G. showed that oil-soluble contrast modulates dendritic cell and regulatory T-cell profiles in the peritoneal cavity in an in vivo and in vitro study [12]. Multiple studies have shown that oil-soluble contrast agents are involved in endometrium immunoregulation. Endometrial slides from animal research have shown that the number of CD205^+^ dendritic cells reduces and that the number of CD1^+^ dendritic cells increases following oil-soluble contrast medium infusion; these changes in uterine dendritic cells may lead to a dampened immune response [13]. Oil-soluble contrast also increased the number of uterine natural killer cells in the endometrium in four women [6]. Our previous studies showed that intrauterine bathing with oil medium reduced inflammation of the endometrium in a rat model with chronic endometritis (CE) by regulating Th1/Th2-type cytokine towards Th2 cells [14].

However, most current research primarily focuses on analyzing the impact of oil-soluble contrast used in HSG, typically conducted during the follicular phase. Few studies examine its effect on the endometrium during the window of implantation (WOI). The status of the endometrium in the WOI plays a vital role in establishing and maintaining normal pregnancy [15]. A comprehensive analysis of the alterations in endometrial immunoregulation during the WOI is essential for a more thorough understanding of how oil-soluble contrast improves fertility.

In addition, insufficient and excessive endometrial inflammatory responses and maternal immune responses during the WOI are proposed to lead to implantation failure, including recurrent implantation failure (RIF) [15].In 2023, the ESHRE Working Group developed the latest recommendation for tailoring recognition of RIF to the specific context of each patient; RIF poses a challenge in the field of Assisted Reproductive Technology (ART) clinics. Although some interventions are available in clinical practice, they are often implemented without a clear biological rationale or evidence of benefit [16]. It is crucial to investigate and validate the effectiveness of more personalized interventions. While the oil-soluble medium has been demonstrated to enhance pregnancy outcomes, there is limited research on its application in treating RIF. An RCT involving 11 women who had a history of RIF in previous in vitro fertilization (IVF) treatments, and the results indicated that administering lipiodol before transferring fresh embryos did not provide any discernible benefits for these women [17]. This trial involved a fresh transplant cycle, and it is known that higher estrogen levels during fresh embryo transfer may impact pregnancy outcomes. However, there is limited research on the effects of frozen-thawed embryo transfer (FET) cycles. Little attention is given to the thickness changes and immunohistological impact on the endometrium during WOI regarding oil-soluble contrast bathing.

Given the reasons mentioned above, there is an apparent necessity for a descriptive study to compare the histological changes of the endometrium and pregnancy outcomes related to oil-soluble contrast in women with RIF. We conducted a descriptive survey of RIF patients undergoing FET after intrauterine bathing with oil-soluble contrast instead of studying the effect of oil-soluble contrast after routine HSG surgery. Endometrial biopsy was performed seven days after ovulation to observe histological changes during the WOI before and after oil-soluble contrasts bathing in patients with RIF in FET cycles. This study’s findings will complement the therapeutic mechanisms of oil-soluble contrast bathing to provide a new approach for treating patients with RIF.

## Materials and methods

Before initiating this prospective, descriptive pilot study, the Ethics Committee of the Affiliated Hospital of Youjiang Medical University for Nationalities and the First Affiliated Hospital of Guangxi Medical University approved this work.

### Patients

This prospective, descriptive pilot study was conducted at the reproductive medicine centers of the First Affiliated Hospital of Guangxi Medical University and the Affiliated Hospital of Youjiang Medical University between December 18, 2019, and December 30, 2020. The study enrolled patients under the age of 40 who had undergone a minimum of four high-quality embryo transfers during three fresh or frozen cycles but were unable to achieve a clinical pregnancy, and they also had available high-quality embryos for further treatment [18]. A convenience sample of thirty-four patients with RIF were enrolled. The inclusion criteria were as follows: women aged 20–40 years with spontaneous menstrual cycles; women who had undergone three or more transfer cycles, cumulative transplantation of at least four high-quality cleavage-stage embryos or three high-quality blastocysts without clinical pregnancy; and high-quality frozen embryos that were still available for implantation, with the transferred embryos reaching a morphological grade of 622, 3BB or better, according to the Gardner scoring criteria. Patients were excluded from participation if they were unable to complete oil-soluble contrast bathing, endometrial biopsy before and after bathing, or embryo transfer within four menstrual cycles after bathing; had severe uterine adhesions, a septum uterus, or uterine leiomyoma; had hyperthyroidism or hypersensitivity to iodine; had severe heart and lung disease, uncontrolled hypertension, or diabetes; had a body mass index (BMI) ≥ 28; had pregnancy-related severe diseases, such as luteal and thyroid dysfunction; or had any conditions that were unstable or unlikely to comply with pregnancy; and couples with chromosome abnormalities or males with very weak sperm.

### Oil-soluble contrast bathing and endometrial biopsy

Uterine bathing with 5 ml of ethiodized poppyseed oil (H20160011, Hengrui, China) was carried out 3–7 days after menstrual bleeding cessation via a balloon uterine catheter or an intrauterine insemination catheter. During the luteal phase, both before and after the uterine bathing cycle, an endometrial biopsy was conducted using a Pipelle sampler. Precisely, ultrasound monitored ovulation, and the endometrial biopsy was performed seven days after ovulation.

### Endometrial preparation protocol for FET

Natural cycles, hormone replacement therapy (HRT) cycles, and gonadotropin-releasing hormone agonist (GnRHa) pretreatment followed by HRT (GnRHa-HRT) cycles were the three protocols for FET. Thawing transfer with cleavage-stage or blastocyst-stage embryos was carried out 4 and 6 days following ovulation, respectively, for the participants with natural cycles. Women undergoing HRT cycles were administered 4–6 mg of estradiol between Days 2 and 4 of the menstrual cycle. Endometrial thickness was assessed by vaginal ultrasound, and the estradiol dosage was increased to 8 mg/d if necessary. When endometrial thickness reaches more significant than 7 mm, patients received a daily injection of 60 mg of progesterone (XianJu Pharma, China) and daily vaginal administration of 400 mg of progesterone in soft capsules (Utrogestan, Laboratoires Besins International, France). During the GnRHa-HRT cycles, patients were administered a 3.75 mg GnRHa injection (Diphereline, Ipsen Pty Ltd, France) on the second day of their menstrual cycle, followed by the commencement of the HRT protocol on Day 28. Cleavage- and blastocyst-stage FET, respectively, commenced at 4 and 6 days after progesterone administration, and progesterone was given until a pregnancy test was carried out.

### Immunohistochemistry (IHC)

Briefly, slides were placed in sodium citrate buffer and then heated for 5 min for antigenic retrieval. The slides were washed three times for 3 min with PBS. The prepared 3% hydrogen peroxide was added dropwise to the tissue and then incubated at room temperature for 15 min. The sections were incubated with goat serum at room temperature for 30 min. The slides were incubated with the primary antibodies against CD56 (Abcam 237708, 1:1500), CD138 (Abcam 128936, 1:1500), ανβ-3 (ZENBIO 381872, 1:3000), and Homeobox 10 **(**HOXA10) antibodies (BIOSS-bs2502R, 1:3000) overnight at 4 °C and then with secondary antibody at room temperature.

CE was diagnosed if at least one of the 30 randomly selected HPFs had five or more CD138-positive plasma cells in the endometrial stroma [19]. Five HPFs were randomly chosen, and the number of CD138-positive cells in each field was calculated for 5 fields. The same method was used to calculate the number of CD56-positive cells in the endometrial stroma. Quantitative analysis of endometrial HOXA10 and ανβ-3 expressions according to the H-score was performed as previously described [20].

### Outcomes and definitions

The following pregnancy outcomes were evaluated: biochemical pregnancy, clinical pregnancy, ongoing pregnancy, live birth, and early miscarriage rate. A biochemical pregnancy was described as an elevated serum HCG level (> 10 IU/L). A transvaginal ultrasound scan confirmed clinical pregnancy 7 weeks after a biochemical pregnancy was diagnosed. Ongoing pregnancy is referred to as maintaining pregnancy for at least 20 weeks. A live birth was the delivery of any newborn after a gestation period of 28 weeks or more. Early miscarriage was defined as the natural termination of the embryo or fetus before 12 weeks of gestation.

### Data collection

We collected data on the following baseline characteristics: age, maternal BMI, infertility duration, embryo transfer cycles, gravidity, time from bathing to FET, and type of infertility. The clinical data of the two FET cycles, from the last cycle before oil-soluble contrast bathing to the first cycle after bathing, endometrial thickness, and endometrial type on the first day of progesterone administration, and data of pregnancy outcomes were collected.

### Statistical analysis

All data analyses were carried out with SPSS Statistics version 26.0. Continuous data for normal distribution were presented as the mean ± SD, Median, and range were used to describe continuous variables for non-normal distribution, and frequencies and percentages were used for categorical variables. The data before and after oil-soluble contrast bathing were evaluated using a paired Student’s *t* test or Wilcoxon rank sum test when variables were not normally distributed. *P* values < 0.05 were considered to indicate statistical significance.

## Results

### Study population and baseline characteristics

**Fig. 1:**
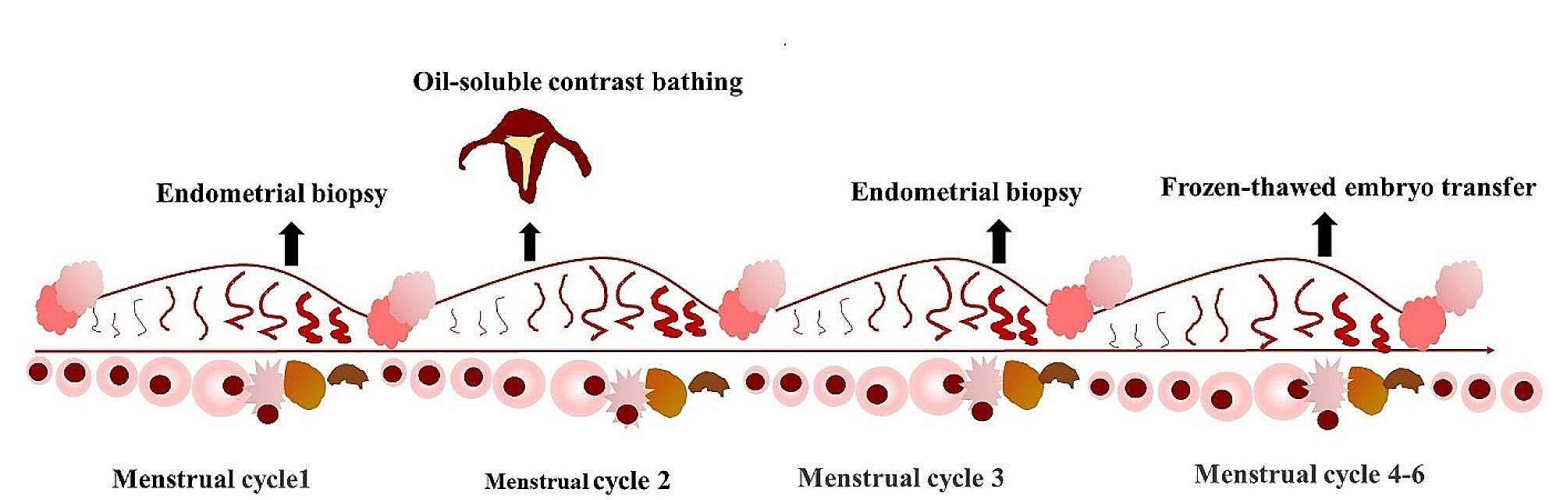
Flow of the study. Endometrial biopsy was carried out in the luteal phase of menstrual cycle 1. Uterine bathing with ethiodized poppyseed oil was carried out 3–7 days after the cessation of menstrual bleeding in the menstrual cycle 2, an endometrial biopsy was carried out once more in the menstrual cycle 3. Freeze-thaw embryo transfer was performed during the menstrual cycle 4–6

The flow of the study is illustrated in Fig. 1. According to the inclusion and exclusion criteria, thirty-four patients were enrolled, of whom seven refused the first endometrial biopsy and were excluded. Four patients who did not complete oil-soluble contrast bathing were also excluded, as were ten who refused the second endometrial biopsy after bathing. One patient who did not receive FET after bathing was excluded. Twelve women were ultimately included in the study analysis. Ten patients were treated at the Reproductive Medical Center, the First Affiliated Hospital of Guangxi Medical University, and two at the Affiliated Hospital of Youjiang Medical University for Nationalities. The median age was 32.5 years (range 27–40 years). The median maternal BMI was 22.20 kg/m² (range 18.89–27.37 kg/m²), and the median infertility duration was 5.5 years (range 2–16 years). The median number of embryo transfer cycles was 3 (range 3–8 cycles), and the median gravidity was 0 (range 0–4). All 12 patients underwent oil bathing smoothly and received endometrial biopsy before and after bathing, and no adverse reactions occurred. Participants spent a median of 72 days (range 52–167 days) from oil bathing to FET (Table 1).

**Table 1:** Baseline characteristics

Variables	Median (range)	mean ± SD
	(*n* = 12)	(*n* = 12)
Age (years)	32.5(27–40)	33.33 ± 4.22
Maternal BMI (kg/m²)	22.2(18.89–27.37)	22.05 ± 2.85
Infertility duration (years)	5.5(2–16)	
Embryo transfer cycles (cycles)	3(3–8)	
Gravidity (numbers)	0.5(0–4)	
Time from bathing to FET (days)	72(52–167)	
Type of infertility	n (%)	
Primary infertility	5(41.7)	
Secondly infertility	7(58.3)	

Abbreviations: BMI: Body Mass Index; FET: frozen-thawed embryo transfer

### Impact on endometrium

**Fig. 2:**
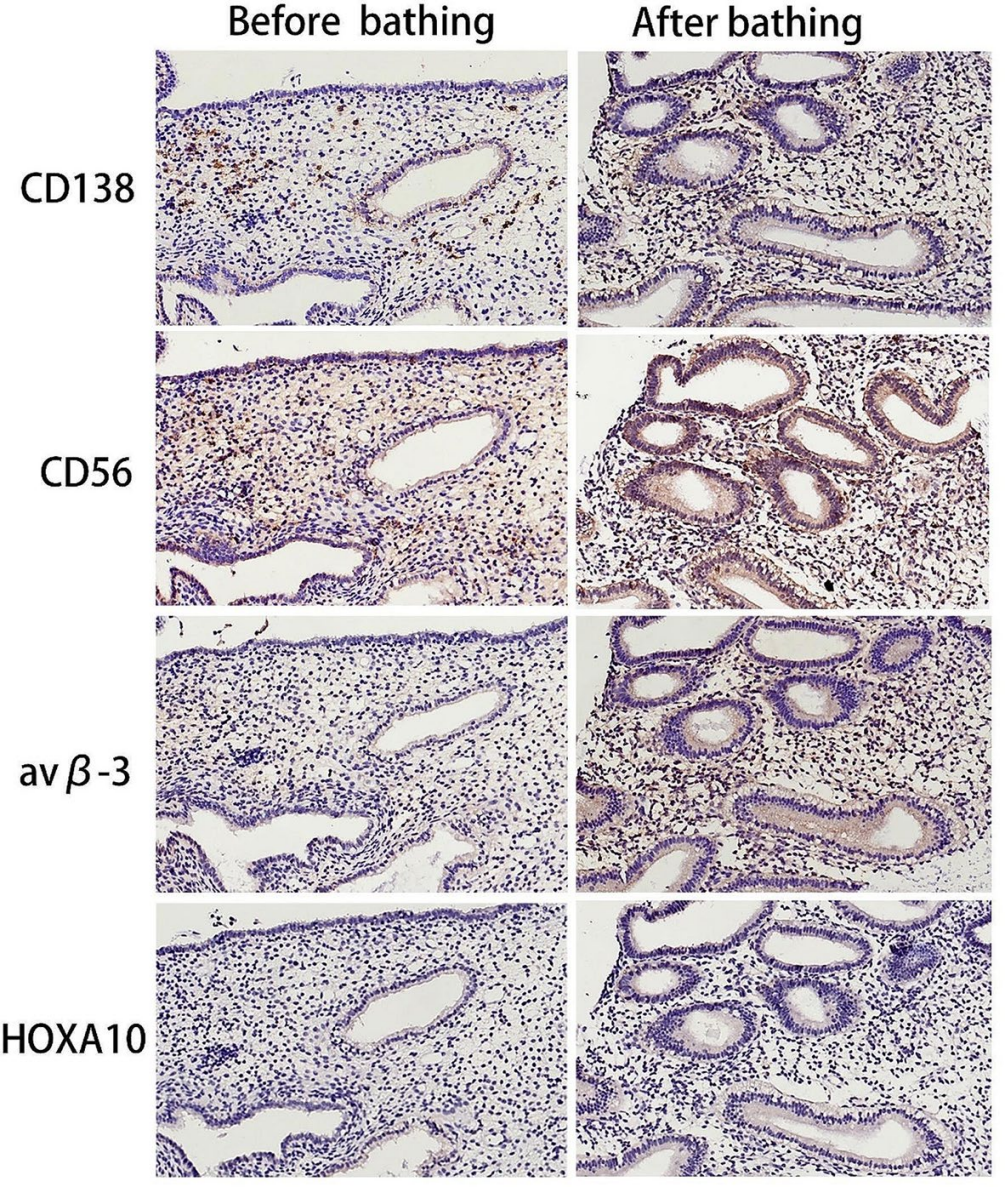
Oil-soluble contrast bathing decreased the number of CD138-positive cells and upregulated expression of endometrial receptivity marker αvβ-3 in RIF patients. No consistent changes were found in the expression of CD56 and HOXA10 in the endometrium after oil-soluble contrast bathing

**Fig. 3:**
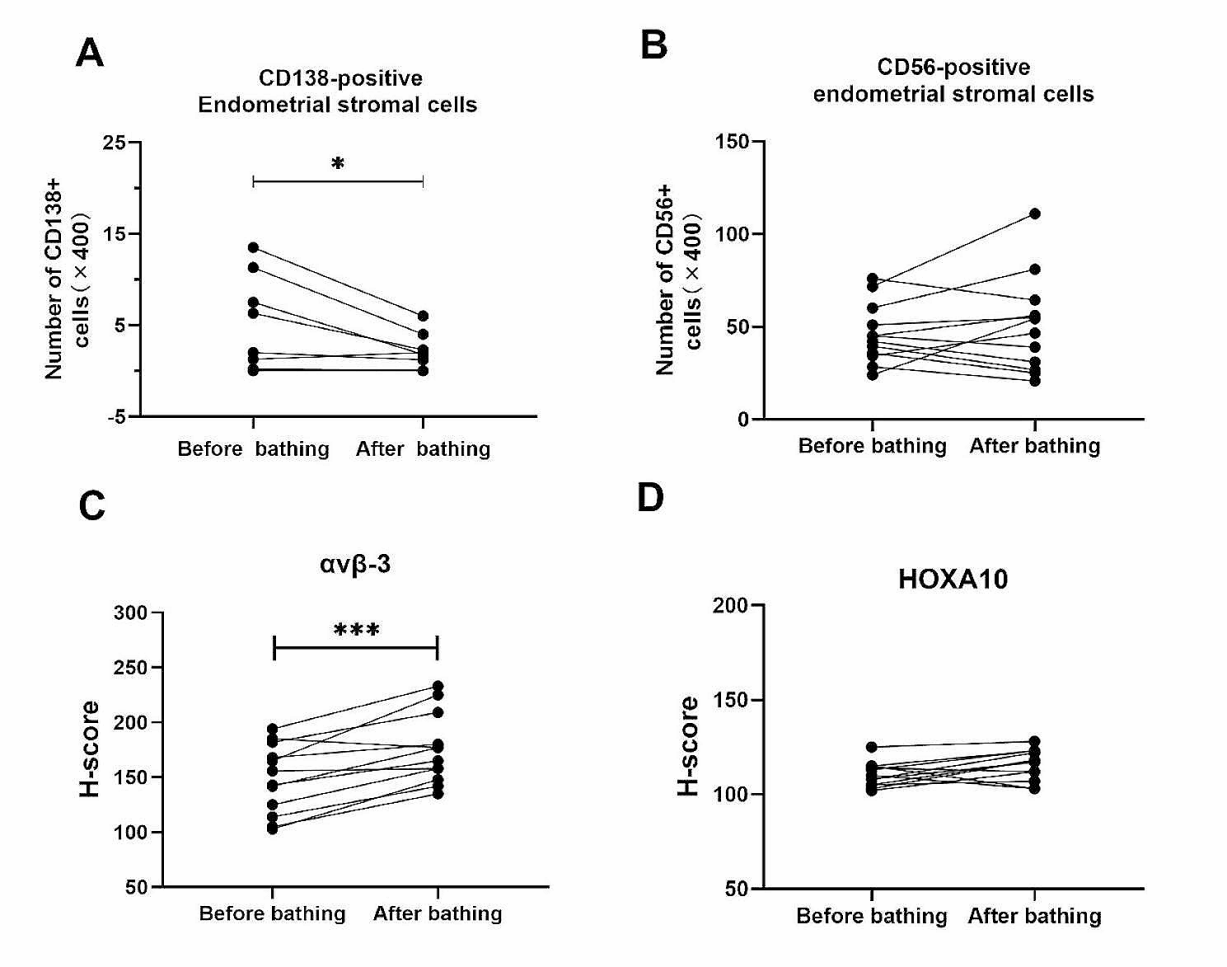
Quantitative comparative analysis of immunohistochemical images in patients with RIF. After oil-based bathing, the number of CD138-positive cells decreased, ^*^P < 0.05, and the H-score of avβ-3 in epithelial and glandular epithelial cells was statistically significantly increased, ^***^P < 0.001. (A–D) Bathing with oil-soluble contrast reduced CD138-positive cells and increased avβ-3 H-score in RIF patients

Before oil-soluble contrast bathing, in 4 of 12 patients diagnosed with CE, the CD138 positivity rate was 33.33%. After oil-soluble contrast bathing, CD138-positive endometrial stromal cells decreased from 0.75 (range 0–13.5) to 0.65 (range 0–6), *P* = 0.035. Expressions of endometrial receptivity marker ανβ-3 in the epithelial and glandular epithelium of the endometrium were upregulated after bathing, and the H-score of ανβ-3 significantly increased (148.50 ± 31.63 vs.175.58 ± 31.83, *P* < 0.001). However, no consistent changes were found in expression of CD56 and HOXA10 in the endometrium after oil-soluble contrast bathing, in the number of CD56-positive cells (46.09 ± 16.19 vs.49.92 ± 26.05, *P* > 0.05), or the H-score of HOXA10 (110.25 ± 6.53 vs.115.50 ± 8.17, *P* > 0.05) (Figs. 2 and 3; Table 2).

**Table 2:** Comparison of endometrial histology before and after oil-soluble contrast bathing

Variables	Before bathing	After bathing	
	(*n* = 12)	(*n* = 12)	
Endometrium thickness (mm, mean ± SD)	8.90 ± 1.45	10.11 ± 1.98^**^	
Endometrial classification			
A, n (%)	6(50%)	8(66.7%)	
B, n (%)	6(50%)	4(33.3%)	
C, n (%)	0	0	
Number of CD138^+^endometrial stromal cells, Median (range)	0.75(0-13.5)	0.65(0–6)^*^	
Number of CD56^+^endometrial stromal cells (mean ± SD)	46.09 ± 16.19	49.92 ± 26.05	
H-score of avβ-3 in epithelial and glandular epithelial cells (mean ± SD)	148.50 ± 31.63	175.58 ± 31.83^***^	
H-score of HOXA10 in epithelial and glandular epithelial cells (mean ± SD)	110.25 ± 6.53	115.5 ± 8.17	

*p < 0.05, **p < 0.01, ***P < 0.001; The average number of CD138 and CD56 positive cells were counted in 5 nonoverlapping random stromal areas visualized at 400-fold magnification

### FET cycles and pregnancy outcomes

Endometrial thickness on the first day of progesterone administration in FET cycles increased significantly after oil-soluble contrast bathing (8.90 ± 1.45 vs. 10.11 ± 1.98 mm, *P* = 0.005), and there was no difference in endometrial type between two FET cycles in the same patient around the time of oil-soluble contrast bathing (Table 2). All twelve patients with RIF underwent FET within 4 menstrual cycles after oil-soluble contrast bathing. Five patients had biochemical pregnancies (5/12, 41.67%), four patients had clinical pregnancies (4/12, 33.33%), one patient had a miscarriage at 9 weeks of pregnancy (1/12, 8.3%), and three patients achieved ongoing pregnancies as well as live births after oil-soluble contrast bathing (3/12, 25%) (Table 3).

**Table 3:** Pregnancy outcomes after oil-soluble contrast bathing

Variables	Oil-soluble contrast bathing	
	(*n* = 12)	
Biochemical pregnancy rate, n (%)	5(41.67)	
Clinical pregnancy rate, n (%)	4(33.33)	
Ongoing pregnancy rate, n (%)	3(25)	
Live birth rate, n (%)	3(25)	
Early miscarriage rate, n (%)	1(8.3)	

## Discussion

This descriptive study suggests that women with unexplained RIF who underwent oil-soluble contrast medium bathing experienced increased endometrial thickness, reduced CD138 expression, and upregulation of ανβ-3 expression during the WOI. The clinical pregnancy and live birth rates after bathing were 33.33% and 25%, respectively. To our knowledge, this is the first descriptive study to describe the immunohistological impact before and after oil-based contrast bathing in the WOI, and limited data exist regarding the effects of oil-based contrast bathing in women with RIF. In 2019, a randomized trial conducted in New Zealand and Pune, India, by Reilly S. J. et al. showed the effects of oil-soluble contrast on the fertility of 11 patients with RIF. For women with RIF who previously underwent IVF and received Lipiodol via HSG and fresh embryo transfer from the IVF cycle, there was no difference in the live birth rates from pregnancies within 6 months [17]. The study exclusively focused on clinical pregnancy outcomes in fresh embryo transfer and did not include an analysis of the endometrium. Unlike their research, our study focused on FET cycles without HSG, and the endometrium and hormone levels in FET cycles were closer to those in the natural physiological state, effectively eliminating the influence of the high estrogen levels caused by the fresh embryo transfer cycle. In this study, self-control showed a clinical pregnancy rate of 33%. Interestingly, after oil-based contrast bathing, the up-regulated expression of receptivity markers avβ-3 and CE remission in the WOI’s endometrium were observed. The results provide preliminary histological confirmation of the regulatory effect of oil-soluble medium on the endometrial microenvironment in women with RIF.

Various factors, such as advanced maternal age and a range of maternal or embryo-related factors, may be responsible for RIF. Uterine anatomic abnormalities, thrombophilia, CE, and immunological factors are among the maternal factors [16, 18, 21]. CE involves a continuous inflammation of the endometrium. The incidence rate of CE among women who are experiencing infertility is between 2.8 and 56.8%, whereas in patients with RIF, it ranges from 14 to 67.5%. CE is connected to alterations in the immune microenvironment in the endometrium of patients with RIF [21]. Moreover, it may adversely affect endometrial receptivity, the embryo implantation process, and the individual WOI. In this study, four women were diagnosed with CE before oil-soluble contrast bathing, and the number of CD138-positive cells significantly decreased after bathing. To begin with, iodine in oil-soluble contrast bathing has potent bactericidal effects on restoring damaged endometrium [22]. Accordingly, oil-soluble contrast bathing may have toxic effects on normal endometrial cells and can cause edema of endometrial stromal cells [14]. These effects may restrain local chronic inflammation in the endometrium. Additionally, the weak immune response and regulation of dendritic cells may be related to the suppression of inflammation. CE has detrimental effects on the individual WOI, leading to embryo-endometrial asynchrony [23]. It is hypothesized that this study’s reduction of CE during the WOI could potentially enhance embryo-endometrial synchrony.

Interestingly, no consistent changes were found in the expression of CD56 in the endometrium after oil-soluble contrast bathing [3]. These results differ from those of Johnson NP [13], who reported increased uterine natural killer cells in the endometrium after lipiodol flushing. The uNK cell population in the endometrium varies during the menstrual cycle and is affected by hormonal factors. Additionally, there were no discernible differences in uNK cell levels in the endometrium of patients with recurrent pregnancy loss [24]. We hypothesize that discrepancies in the research subjects, timing of endometrial sample collection, and the limited sample size may have contributed to the inconsistent findings. Future studies with larger samples based on WOI are warranted.

Multiple studies have confirmed that oil-based media by HSG significantly improves fertility outcomes compared to water-based media [1–3]. Our study used a half-dose of oil-soluble contrast for HSG for uterine bathing, which was different from conventional HSG, and the properties of oil-based media on the endometrium of the WOI were a key concern. Lier M.C.I. performed an RCT in endometriosis patients in which gel was injected into the uterus before IVF/intracytoplasmic sperm injection (ICSI) treatment, and they did not find a favorable effect of uterine infusion [25], which suggests that the fertility-enhancing effects may be related to the properties of oil-based media. Lipiodol and ethiodized poppyseed oil are clinically available oil-based mediums, and *Papaver somniferum seeds*, the primary raw materials in oil-based media agents, contain natural opium [26]. Oil contrast agents may act on endometrial opioid receptors, improving endometrial receptivity [27]. In addition, endogenous opioid peptides regulate pregnant physiology and perform functions by binding to G-protein coupled receptors, including the mu opioid receptor (MOR) [28], delta opioid receptor, and kappa opioid receptor in the human endometrium. The expression of these receptors fluctuates throughout women’s menstrual cycle, with an increase during the proliferative phase and a decrease during the secretory phase [29, 30]. . Indicating the crucial function of opioid receptors in reproductive events. In this study, oil-soluble contrast bathing was conducted 3–7 days after the cessation of menstrual bleeding. The presence of natural opium in oil-soluble contrast may have an impact on endogenous opioid peptides, but further experimental verification is required.

On the other hand, the active ingredient of poppy seed oil contains up to 98% polyunsaturated fatty acids (PUFAs) [31]. In recent years, multiple studies have confirmed that PUFAs affect several reproductive processes via immune regulation, endometrial decidualization regulation, producing sex hormones, and endometrium receptivity [32]. In this study, the increased expression of ανβ-3 in the endometrium and thickened endometrium suggests that oil-soluble contrast may enhance endometrial receptivity. PUFAs present in oil-based contrast agents may contribute to the increased receptivity of the endometrium.

As an invasive operation, the risks associated with oil-soluble contrast cannot be ignored, and emerging evidence suggests that oil-soluble contrast in HSG may also impact maternal and neonatal thyroid function [33]. The oil-soluble contrast used in HSG has a high iodine content and long half-life, leading to potential iodine excess. Mild but persistent subclinical hypothyroidism frequently occurs with late-onset hyperthyroidism, which develops in 38% of participants, and by week 4, 5% of participants develop later-onset hyperthyroidism [34]. Preconception exposure to oil-based contrast in HSG might exert a far-reaching impact on maternal and offspring iodine status. Among 70 of 425 pregnant women with preconception ethiodized-oil HSG, iodine excess was initially confirmed in 38 (54.3%), and the iodine concentration in maternal breast milk and neonatal urine was also higher than that in normative data [35]. In 2023, a retrospective study by Mathews DM et al. revealed that while preconception oil-soluble contrast did not lead to neonatal hypothyroidism, higher maternal peak urine iodine concentration levels during pregnancy were linked to elevated neonatal serum thyroid-stimulating hormone levels [36]. Furthermore, the known adverse effects of pain, intravasation, oil embolism, and lipogranuloma formation are associated with oil-soluble contrast in HSG [34]. Some complications occur covertly. A two-year retrospective survey in western Australia recently revealed an intravasation rate of 7.1% in HSG; among these cases, only 45% were reported, and 32% were graded [37]. All our patients who received oil-based intracavitary bathing showed no occurrence of the above-mentioned adverse reactions. However, given the significance of these risks, it is imperative to meticulously deliberate on oil-soluble contrast bathing. This entails a thoughtful selection of the target treatment population, proper techniques and methods of bathing, and dosage of the oil-soluble contrast. A recent paper reported that ultrasound-­guided oil-soluble contrast HSG is safe [38]. Notably, the dosage of the oil-soluble contrast agent utilized for this study was 5 ml, representing a 50% reduction compared to the standard amount typically employed in traditional HSG procedures. A lower dosage of oil-soluble contrast medium reduces the risk of iodine excess and intravasation and minimizes the risk of fetal or neonatal thyroid dysfunction [39]. Based on our findings, a 5 ml oil-soluble contrast agent can effectively bathe the uterus while reducing the risk of iodine excess and intravasation.

Oil-soluble contrast bathing without X-ray irradiation is a simple procedure that can be performed with a low dose of oil-soluble contrast to reduce the risk of iodine excess, intravasation, and medication costs. Studies have demonstrated that oil-soluble contrast can significantly enhance pregnancy outcomes in individuals with endometriosis and unexplained fertility [4, 6, 7]. The latest retrospective cohort study conducted in China by our research group in 2024 showed oil-soluble contrast agents used in HSG for women with endometriosis-related infertility are associated with higher clinical pregnancy rates and live birth rates [7]. Furthermore, this study provides preliminary verification of the effectiveness of oil-soluble contrast in alleviating inflammation in CE and improving endometrial thickness and receptivity. Therefore, oil-soluble contrast bathing may present a promising application prospect for individualized treatment in patients with reproductive disorders such as RIF, endometriosis, thin endometrium, and CE. However, it is essential to note that intrauterine bathing with oil-soluble contrast is an invasive procedure and should be strictly controlled in clinical application. There is also a risk of transient thyroidism associated with this procedure, so thyroid dysfunction should be ruled out before interventions.

The main limitation of our study is that it was a pilot study with a small sample size. Due to the limited size of our study population and the low completion rate of two endometrial biopsies before and after bathing, it is challenging to increase the sample size as many enrolled patients were excluded. In addition, the non-RCT design of our study may compromise the scientific and rigorous nature of our results. Further well-designed RCTs with larger and more robust sample sizes are imperative.

## Conclusion

This study demonstrates that oil-soluble contrast bathing can eliminate CD138-positive plasma cells and improve the thickness of the endometrium and endometrial receptivity in RIF patients. This impact of endometrium may contribute to enhanced fertility in women with RIF, and oil-based contrast bathing may be a new approach for improving pregnancy outcomes in the RIF population for FET cycles. Further research and well-designed RCTs with larger sample sizes are needed to determine the therapeutic effect and mechanism of oil-based contrast on RIF patients.

## Data Availability

On reasonable request, the corresponding author will provide the datasets used and analyzed during the current work.

## Abbreviations

HSG: Hysterosalpingography
CE: Chronic endometritis
WOI: Window of implantation
RIF: Recurrent implantation failure
RCT: Randomized controlled trials
IVF: In vitro fertilization
FET: Frozen-thawed embryo transfer
BMI: Body mass index
HRT: Hormone replacement therapy
IHC: Immunohistochemistry
HOXA10: Homeobox 10
GnRHa: Gonadotropin-releasing hormone agonist
ICSI: Intra cytoplasmatic sperm injection
MOR: Mu opioid receptor
PUFAs: Polyunsaturated fatty acids
